# 2-Iodo-5-nitro­thio­phene

**DOI:** 10.1107/S1600536810017356

**Published:** 2010-05-22

**Authors:** Xing Yan Xu, Gang Huang, Xiang Chao Zeng, Fang Hu

**Affiliations:** a Department of Chemistry, Jinan University, Guangzhou, Guangdong 510632, People’s Republic of China

## Abstract

The title compound, C_4_H_2_INO_2_S, was synthesized by nitration of iodo­thio­phene with acetyl nitrate. The molecule is essentially planar, withthe nitro group tilted by 1.78 (19)° and the iodine atom displaced by 0.0233 (2) Å with respect to the thiophene ring. In the crystal structure, adjacent mol­ecules are linked through weak I⋯O inter­actions [3.039 (2)Å], forming chains extending along the *b* axis.

## Related literature

For the bioactivity of thio­phene derivatives, see: Wilson *et al.* (2010 ▶); Rudra *et al.* (2007 ▶); Altman *et al.* (2008 ▶); Morley *et al.* (2006 ▶).
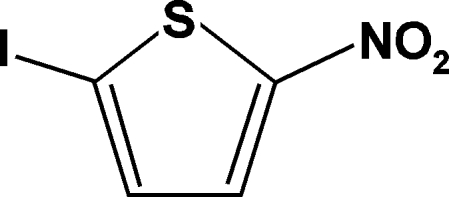


## Experimental

### 

#### Crystal data

C_4_H_2_INO_2_S
*M* *_r_* = 255.03
Monoclinic, 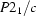

*a* = 9.195 (2) Å
*b* = 9.727 (2) Å
*c* = 7.6714 (17) Å
β = 105.043 (4)°
*V* = 662.6 (2) Å^3^
*Z* = 4
Mo *K*α radiation
μ = 5.07 mm^−1^
*T* = 110 K
0.48 × 0.29 × 0.08 mm

#### Data collection

Bruker SMART 1K CCD area-detector diffractometer
Absorption correction: multi-scan (*SADABS*; Sheldrick, 1996 ▶) *T*_min_ = 0.195, *T*_max_ = 0.687
3264 measured reflections
1419 independent reflections
1294 reflections with *I* > 2σ(*I*)
*R*_int_ = 0.022

#### Refinement

*R*[*F* ^2^ > 2σ(*F* ^2^)] = 0.025
*wR*(*F* ^2^) = 0.065
*S* = 1.09
1419 reflections
82 parameters
H-atom parameters constrained
Δρ_max_ = 1.65 e Å^−3^
Δρ_min_ = −1.07 e Å^−3^

### 

Data collection: *SMART* (Bruker, 1999 ▶); cell refinement: *SAINT-Plus* (Bruker, 1999 ▶); data reduction: *SAINT-Plus*; program(s) used to solve structure: *SHELXS97* (Sheldrick, 2008 ▶); program(s) used to refine structure: *SHELXL97* (Sheldrick, 2008 ▶); molecular graphics: *SHELXTL* (Sheldrick, 2008 ▶); software used to prepare material for publication: *SHELXTL*.

## Supplementary Material

Crystal structure: contains datablocks I, global. DOI: 10.1107/S1600536810017356/rz2440sup1.cif

Structure factors: contains datablocks I. DOI: 10.1107/S1600536810017356/rz2440Isup2.hkl

Additional supplementary materials:  crystallographic information; 3D view; checkCIF report

## Figures and Tables

**Fig. 1.**
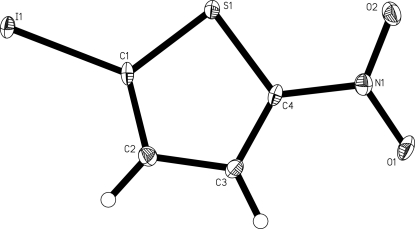
The molecular structure of the title compound, with the atom-numbering scheme. Displacement ellipsoids are drawn at the 30% probability level.

**Fig. 2.**
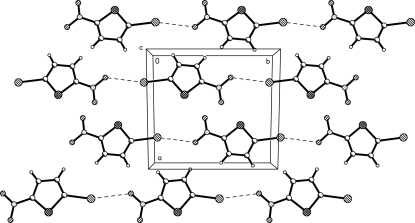
Crystal packing of the the title compound viewed along the c axis. Dashed lines indicate weak intermolecular interactions.

## supplementary crystallographic information

### Comment

Many thiophene derivatives show important bioactivities and are employed as antibacterial (Morley *et al.*, 2006), as inhibitors of Janus kinases (Wilson *et al.*, 2010), in the identification of RBx 8700 (Rudra *et al.*, 2007) and in the treatment of myeloproliferative disorders and cancers (Altman *et al.*, 2008). This is the reason they have attracted our interest.

The molecule of the title compound (Fig. 1) is essentially planar, with the nitro group tilted by 1.78 (19)° and the iodine atom displaced by 0.0233 (2) Å with respect to the thiophene ring. In the crystal structure, adjacent molecules are linked through weak I···O interactions to form chains extending along the *b* axis. (Fig. 2).

### Experimental

Iodothiophene (6.5 g, 31 mmol) dissolved in 10 ml of acetic anhydride was introduced into a round flask, provided with a stirrer and a cooling device. Acetyl nitrate was added dropwise in forty-five minutes and the temperature was kept under 273 K. When the addition was over, the mixture was stirred for half an hour continuously at the same temperature. The nitrating flask was then surrounded with ice and kept in a refrigerator for 24 hours. The product was poured, with stirring, into finely crushed ice. After filtration, the precipitate was collected as a yellow solid. The impure product was dissolved in methanol, pale yellow monoclinic crystals suitable for X-ray analysis (m.p. 348 K, 67% yield) grew over a period of five days on slow evaporation of the solvent at room temperature.

### Refinement

All non-H atoms were refined with anisotropic displacement parameters. The H atoms were positioned geometrically (C—H = 0.95 Å) and refined using a riding model, with *U*_iso_ = 1.2 *U*_eq_(C).

### Crystal data

**Table:**

C_4_H_2_INO_2_S	*F*(000) = 472
*M**_r_* = 255.03	*D*_x_ = 2.556 Mg m^−^^3^
Monoclinic, *P*2_1_/*c*	Melting point: 348 K
Hall symbol: -P 2ybc	Mo *K*α radiation, λ = 0.71073 Å
*a* = 9.195 (2) Å	Cell parameters from 2600 reflections
*b* = 9.727 (2) Å	θ = 2.3–27.0°
*c* = 7.6714 (17) Å	µ = 5.07 mm^−^^1^
β = 105.043 (4)°	*T* = 110 K
*V* = 662.6 (2) Å^3^	Plate, pale yellow
*Z* = 4	0.48 × 0.29 × 0.08 mm

### Data collection

**Table:**

Bruker SMART 1K CCD area-detector diffractometer	1419 independent reflections
Radiation source: fine-focus sealed tube	1294 reflections with *I* > 2σ(*I*)
graphite	*R*_int_ = 0.022
φ and ω scans	θ_max_ = 27.0°, θ_min_ = 2.3°
Absorption correction: multi-scan (*SADABS*; Sheldrick, 1996)	*h* = −11→10
*T*_min_ = 0.195, *T*_max_ = 0.687	*k* = −12→6
3264 measured reflections	*l* = −9→9

### Refinement

**Table:**

Refinement on *F*^2^	Primary atom site location: structure-invariant direct methods
Least-squares matrix: full	Secondary atom site location: difference Fourier map
*R*[*F*^2^ > 2σ(*F*^2^)] = 0.025	Hydrogen site location: inferred from neighbouring sites
*wR*(*F*^2^) = 0.065	H-atom parameters constrained
*S* = 1.09	*w* = 1/[σ^2^(*F*_o_^2^) + (0.0417*P*)^2^ + 0.2502*P*] where *P* = (*F*_o_^2^ + 2*F*_c_^2^)/3
1419 reflections	(Δ/σ)_max_ = 0.001
82 parameters	Δρ_max_ = 1.65 e Å^−^^3^
0 restraints	Δρ_min_ = −1.07 e Å^−^^3^

### Special details

**Table:**

Geometry. All esds (except the esd in the dihedral angle between two l.s. planes) are estimated using the full covariance matrix. The cell esds are taken into account individually in the estimation of esds in distances, angles and torsion angles; correlations between esds in cell parameters are only used when they are defined by crystal symmetry. An approximate (isotropic) treatment of cell esds is used for estimating esds involving l.s. planes.
Refinement. Refinement of *F*^2^ against ALL reflections. The weighted *R*-factor wR and goodness of fit *S* are based on *F*^2^, conventional *R*-factors *R* are based on *F*, with *F* set to zero for negative *F*^2^. The threshold expression of *F*^2^ > σ(*F*^2^) is used only for calculating *R*-factors(gt) etc. and is not relevant to the choice of reflections for refinement. *R*-factors based on *F*^2^ are statistically about twice as large as those based on *F*, and *R*- factors based on ALL data will be even larger.

### Fractional atomic coordinates and isotropic or equivalent isotropic displacement parameters (Å^2^)

**Table:**

	*x*	*y*	*z*	*U*_iso_*/*U*_eq_	
I1	0.742893 (19)	0.45070 (2)	−0.00911 (2)	0.01661 (11)	
S1	0.63408 (8)	0.77403 (7)	−0.09375 (9)	0.01554 (17)	
C1	0.7728 (3)	0.6618 (3)	0.0128 (3)	0.0154 (6)	
O2	0.5694 (3)	1.06507 (19)	−0.1375 (3)	0.0224 (5)	
N1	0.6975 (3)	1.0473 (2)	−0.0419 (3)	0.0173 (5)	
C2	0.9003 (3)	0.7266 (3)	0.1129 (4)	0.0176 (6)	
H2	0.9873	0.6796	0.1805	0.021*	
C3	0.8865 (3)	0.8712 (3)	0.1034 (3)	0.0171 (6)	
H3	0.9627	0.9336	0.1630	0.021*	
O1	0.7865 (3)	1.1411 (2)	0.0229 (3)	0.0257 (5)	
C4	0.7496 (3)	0.9090 (4)	−0.0027 (3)	0.0148 (6)	

### Atomic displacement parameters (Å^2^)

**Table:**

	*U*^11^	*U*^22^	*U*^33^	*U*^12^	*U*^13^	*U*^23^
I1	0.02192 (16)	0.00881 (16)	0.01937 (15)	0.00103 (5)	0.00585 (10)	0.00003 (5)
S1	0.0171 (4)	0.0104 (3)	0.0176 (3)	0.0009 (2)	0.0018 (3)	−0.0006 (2)
C1	0.0216 (14)	0.0082 (14)	0.0184 (14)	0.0040 (10)	0.0087 (11)	0.0022 (9)
O2	0.0232 (12)	0.0178 (10)	0.0254 (11)	0.0056 (8)	0.0047 (9)	0.0048 (8)
N1	0.0215 (15)	0.0148 (13)	0.0163 (12)	0.0016 (9)	0.0060 (11)	0.0011 (8)
C2	0.0165 (14)	0.0168 (13)	0.0196 (14)	0.0021 (10)	0.0046 (11)	0.0006 (10)
C3	0.0176 (14)	0.0132 (13)	0.0201 (14)	−0.0027 (10)	0.0042 (11)	−0.0028 (10)
O1	0.0331 (12)	0.0094 (11)	0.0324 (12)	−0.0033 (9)	0.0044 (10)	−0.0019 (8)
C4	0.0206 (18)	0.0087 (16)	0.0167 (16)	−0.0007 (9)	0.0079 (13)	−0.0021 (8)

### Geometric parameters (Å, °)

**Table:**

I1—C1	2.073 (3)	N1—C4	1.433 (4)
S1—C1	1.716 (3)	C2—C3	1.412 (4)
S1—C4	1.719 (3)	C2—H2	0.9500
C1—C2	1.376 (4)	C3—C4	1.361 (4)
O2—N1	1.227 (4)	C3—H3	0.9500
N1—O1	1.241 (3)		
			
C1—S1—C4	89.32 (14)	C1—C2—H2	124.0
C2—C1—S1	113.2 (2)	C3—C2—H2	124.0
C2—C1—I1	125.1 (2)	C4—C3—C2	110.9 (3)
S1—C1—I1	121.68 (16)	C4—C3—H3	124.5
O2—N1—O1	124.5 (2)	C2—C3—H3	124.5
O2—N1—C4	118.3 (2)	C3—C4—N1	125.9 (3)
O1—N1—C4	117.2 (3)	C3—C4—S1	114.5 (3)
C1—C2—C3	112.0 (3)	N1—C4—S1	119.59 (19)
			
C4—S1—C1—C2	0.2 (2)	O2—N1—C4—C3	−178.6 (2)
C4—S1—C1—I1	179.18 (13)	O1—N1—C4—C3	1.9 (4)
S1—C1—C2—C3	−0.2 (3)	O2—N1—C4—S1	1.5 (3)
I1—C1—C2—C3	−179.22 (17)	O1—N1—C4—S1	−178.06 (17)
C1—C2—C3—C4	0.2 (3)	C1—S1—C4—C3	0.0 (2)
C2—C3—C4—N1	180.0 (2)	C1—S1—C4—N1	179.92 (18)
C2—C3—C4—S1	−0.1 (3)		

## References

[bb1] Altman, M., Christopher, M., Grimm, J. B., Haidle, A., Konrad, K., Lim, J., Maccoss, R. N., Machacek, M., Osimboni, E., Otte, R. D., Siu, T., Spencer, K., Taoka, B., Tempest, P., Wilson, K., Woo, H. C., Young, J. & Zabierek, A. (2008). PCT Int. Appl. WO 2008156726.

[bb2] Bruker (1999). *SMART* and *SAINT-Plus* Bruker AXS Inc., Madison, Wisconsin, USA.

[bb3] Morley, J. O. & Matthews, T. P. (2006). *Org. Biomol. Chem.* **4**, 359–366. 10.1039/b514441h 16391779

[bb4] Rudra, S., Yadav, A., Raja Rao, A. V. S., Srinivas, A. S. S. V., Pandya, M., Bhateja, P., Mathur, T., Malhotra, S., Rattan, A., Salman, M., Mehta, A., Cliffe, I. A. & Das, B. (2007). *Bioorg. Med. Chem. Lett.* **17**, 6714–6719. 10.1016/j.bmcl.2007.10.061 17980588

[bb5] Sheldrick, G. M. (1996). *SADABS* University of Göttingen, Germany.

[bb6] Sheldrick, G. M. (2008). *Acta Cryst.* A**64**, 112–122. 10.1107/S0108767307043930 18156677

[bb7] Wilson, K., De Almeida, G., Haidle, A., Konrad, K., Machacek, M. & Zabierek, A. (2010). PCT Int. Appl. WO 2010005841.

